# MicroRNAs in glioblastoma multiforme pathogenesis and therapeutics

**DOI:** 10.1002/cam4.775

**Published:** 2016-06-10

**Authors:** Amanda Shea, Varsha Harish, Zainab Afzal, Juliet Chijioke, Habib Kedir, Shahnoza Dusmatova, Arpita Roy, Malathi Ramalinga, Brent Harris, Jan Blancato, Mukesh Verma, Deepak Kumar

**Affiliations:** ^1^ Division of Science and MathematicsCancer Research LaboratoryUniversity of the District of ColumbiaWashingtonDistrict of Columbia20008; ^2^ Groton SchoolGrotonMassachusetts01450; ^3^ Department of Neurology and PathologyGeorgetown UniversityWashingtonDistrict of Columbia20057; ^4^ Lombardi Comprehensive Cancer CenterGeorgetown UniversityWashingtonDistrict of Columbia20057; ^5^ Division of Cancer Control and Population SciencesNational Cancer Institute (NCI)National Institutes of Health (NIH)RockvilleMaryland20850

**Keywords:** cancer therapy, microRNA, glioblastoma multiforme

## Abstract

Glioblastoma multiforme (GBM) is the most common and lethal cancer of the adult brain, remaining incurable with a median survival time of only 15 months. In an effort to identify new targets for GBM diagnostics and therapeutics, recent studies have focused on molecular phenotyping of GBM subtypes. This has resulted in mounting interest in microRNAs (miRNAs) due to their regulatory capacities in both normal development and in pathological conditions such as cancer. miRNAs have a wide range of targets, allowing them to modulate many pathways critical to cancer progression, including proliferation, cell death, metastasis, angiogenesis, and drug resistance. This review explores our current understanding of miRNAs that are differentially modulated and pathologically involved in GBM as well as the current state of miRNA‐based therapeutics. As the role of miRNAs in GBM becomes more well understood and novel delivery methods are developed and optimized, miRNA‐based therapies could provide a critical step forward in cancer treatment.

## Introduction

Glioblastoma multiforme (GBM) is the most common and lethal cancer of the brain, with approximately 10,000 newly diagnosed cases each year in the United States 1. Despite recent advances in understanding of its pathogenesis, GBM remains incurable with standard treatment options contributing little to survival time. Currently, GBM has a 5‐year survival rate of only 10% and a median survival time of 15 months following treatment 1, 2. Contributing to its poor prognosis are numerous therapeutic challenges including aggressive growth rates, tumor heterogeneity, drug resistance, and obstacles to drug delivery such as the blood–brain barrier.

In an effort to find novel approaches to GBM treatment, recent studies have focused on molecular phenotyping of GBM subtypes to identify new targets for biomarkers and therapeutics. Of particular interest have been microRNAs (miRNAs), an abundant class of endogenously expressed 18–25 nucleotide noncoding RNAs. These molecules can inhibit gene expression by binding to target messenger RNA (mRNA), thereby inducing translational silencing or degradation based on complementarity to targets 3.

**Figure 1:**
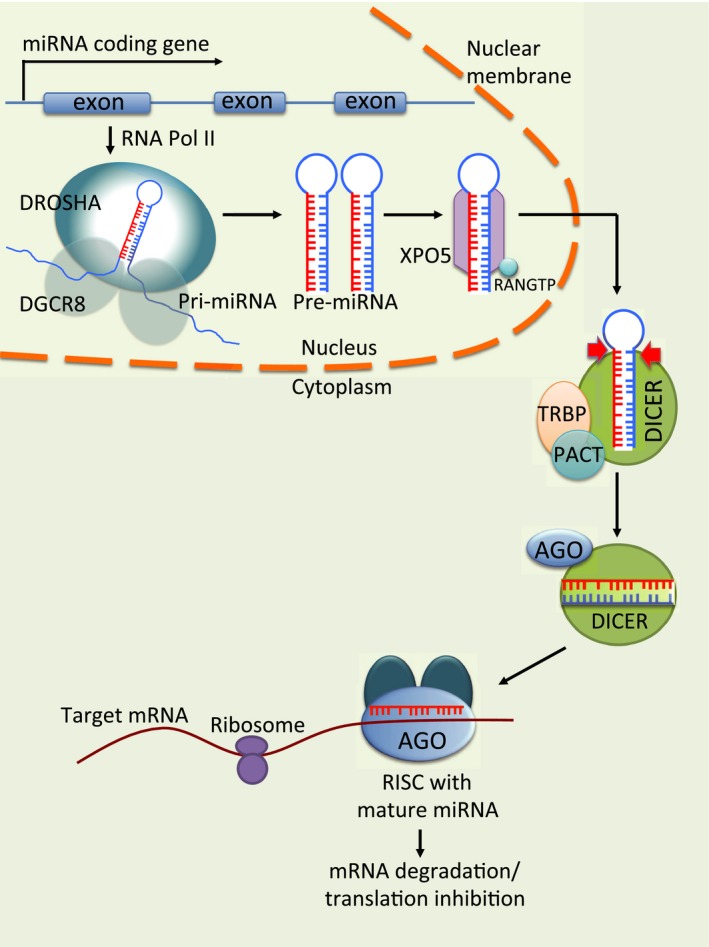
Biogenesis of miRNAs. miRNAs are encoded by nuclear DNA and are transcribed by RNA polymerase II to generate pri‐miRNAs. Pri‐miRNAs then fold into stem‐loop structures via intramolecular base pairing and are cleaved by the microprocessor complex. The resulting long hairpin miRNA precursors, called pre‐miRNAs, are then transported from the nucleus by Exportin‐5. In the cytoplasm, pre‐miRNAs are cleaved by the RNase III enzyme Dicer, to produce miRNA duplexes. The duplexes are then unwound and the guide strands are selected by Argonaute for integration into the RNA‐induced silencing complex (RISC). Within RISC, miRNAs serve as templates for recognizing complementary mRNA molecules, resulting in degradation and/or translational repression of target genes.

miRNAs are encoded by nuclear DNA and are transcribed by RNA polymerase II to generate capped and polyadenylated long primary transcripts, called pri‐miRNAs (Fig. 1) 4. Each pri‐miRNA folds into a stem‐loop structure via intramolecular base pairing 5 and is then cleaved by the microprocessor complex, which consists of Drosha and DGCR8. The product is a 60–120 nucleotide long hairpin miRNA precursor, called pre‐miRNA 6, 7. Pre‐miRNA is actively transported from the nucleus by Exportin‐5 8. In the cytoplasm, the RNase III enzyme Dicer, makes the final cleavage, producing a ~22‐nucleotide miRNA duplex. Association of Dicer with RNA‐binding domain factors, PACT or TRBP, can also produce slightly different‐sized miRNAs, termed isomiRs, which have altered target‐binding specificities 9. After the mature miRNA is formed, the duplex is unwound. While one strand is normally degraded, the other is incorporated into the RNA‐induced silencing complex (RISC). Within RISC, the miRNA serves as a template for recognizing its complementary mRNA molecule 10, most frequently within the 3′ untranslated region. This interaction results in degradation and/or translational repression of target genes.

There are currently 2588 mature human sequences recorded in miRBase, a public miRNA repository (www.mirbase.org, accessed January 2016). Each is predicted to individually regulate hundreds to thousands of mRNAs, depending on cell type, context, and binding of cofactors 11, 12. Further, specific mRNAs can also be modulated by numerous different miRNAs, thus allowing for widespread regulation of gene expression. Indeed, it is estimated that miRNAs regulate up to one‐third of human genes 13, participating in various processes including cell proliferation, differentiation, cell cycle regulation, and apoptosis.

Importantly, miRNAs have also been implicated in various pathological conditions, including cancer. Virtually all tumors display globally abnormal miRNA expression patterns 14, and more than 50% of the human miRNA‐encoding genes are found in fragile chromosomal sites associated with cancer 15. These alterations in miRNA expression can occur through a variety of mechanisms including altered transcriptional regulation, abnormalities in miRNA processing, defects in localization of miRNAs, gene mutations, chromosomal changes, epigenetic aberrations, and alterations in the machinery involved in miRNA biogenesis 16. This frequently results in upregulation of oncogenes and/or downregulation of tumor suppressors, thereby functioning to support carcinogenesis.

### miRNAs in GBM

Many studies have begun to map out the expression profiles and functions of miRNAs in GBM, aiming to garner new insight that can be used to combat this insidious disease. A recent systematic review by Møller et al. 17 found that the most common aberration in miRNA expression with GBM is upregulation. Indeed, 256 miRNAs were found to be significantly overexpressed (notably miR‐10b, miR‐17–92 cluster, miR‐21, and miR‐93) and 95 miRNAs were significantly underexpressed (including miR‐7, miR‐34a, miR‐128, and miR‐137) in GBM as compared to normal brain tissue 17. Moreover, individual miRNAs have been correlated with different glioma stages. Investigating the miRNA expression profiles of WHO grade II gliomas that spontaneously progressed to WHO grade IV GBM, Malkorn et al. 18 identified 12 miRNAs (miR‐9, miR‐15a, miR‐16, miR‐17, miR‐19a, miR‐20a, miR‐21, miR‐25, miR‐28, miR‐130b, miR‐140, and miR‐210) that were upregulated and two (miR‐184 and miR‐328) that were downregulated during progression 18. Other studies have additionally found miRNAs that are differentially expressed in advanced clinical stages of GBM, including miR‐182, which is upregulated and miR‐137, which is downregulated in late stages 19, 20.

Importantly, miRNAs seem to have a predilection for targeting developmental genes, making them fundamental in the regulation of proliferation, differentiation, and apoptosis. In relation to cancer pathobiology, this means that miRNAs impact many of the hallmarks of cancer 21, 22. Indeed, miRNAs have been found to contribute to sustained proliferative signaling and evasion of growth suppressors, resistance to cell death, enabling of replicative immortality, induction of angiogenesis, and activation of invasion and metastasis. Further, miRNAs are also important regulators of drug resistance in GBM. Table 1 provides an overview of miRNAs that are known to participate in GBM, including which pathways they have been found to interact with. In the following sections, we briefly discuss the impact of various miRNAs on these pathways.

**Table 1:** miRNAs associated with Glioblastoma multiforme

miRNA	mRNA targets	Expression change with GBM/ poor prognosis	Role(s) in cancer progression	References
let‐7	NRAS, KRAS, CCND1	Decrease	Proliferation, apoptosis, migration, invasion, chemoresistance	35, 143, 144
miR‐7	EGFR, RAF1, PI3K, FAK, IRS2	Decrease	Survival, proliferation, apoptosis, invasion, angiogenesis	23, 24, 75, 145, 146, 147, 148, 149
miR‐9/miR‐9*	SOX2, PTCH1, FOXP1, CAMTA1	Increase;decrease	Proliferation, stemness, chemoresistance	18, 138, 150, 151, 152, 153, 154
miR‐10a/b	BCL2L11, TFAP2C, CDKN2A, CDKN1A, CSMD, HOXD10, E2F1	Increase	Proliferation, apoptosis, migration, invasion, stemness	72, 148, 149, 155, 156, 157, 158
miR‐15a		Increase		18
miR‐15b	NRP2, CCND1	Decrease	Proliferation, apoptosis, invasion, angiogenesis	159, 160, 161
miR‐16	BMI1, NFKB1, BCL2, ZYX	Decrease;increase 18	Proliferation, apoptosis, migration, invasion, angiogenesis	18, 162, 163, 164
miR‐17	CAMTA1, PTEN, MDM2	Increase	Survival, proliferation, migration, invasion, stemness, chemoresistance, stress response	18, 84, 154, 165, 166
miR 17–92 cluster	CTFG	Increase	Proliferation, apoptosis, stemness	167
miR‐18a/18a*	NEO1, DLL3, CTGF, SMAD3	Increase	Proliferation, apoptosis, migration, invasion, stemness	168, 169, 170
miR‐19a/b	PTEN	Increase	Survival, proliferation	18, 171
miR‐20a	TIMP2	Increase	Invasion	18, 84, 172
miR‐21	PDCD4, IGFB3, FBXO11, ANP32A, SMARCA4, LRRFIP1, HNRPK, TP63, RECK, TIMP3, TPM1, FASLG, SPRY2	Increase	Survival, proliferation, apoptosis, migration, invasion, chemoresistance	18, 37, 38, 39, 40, 46, 47, 69, 70, 90, 149, 173, 174, 175, 176, 177, 178
miR‐23b	PTK2B	Decrease	Migration, invasion	179
miR‐24	ST7L	Increase	Proliferation, apoptosis, invasion	180
miR‐25	CDKN1C, NEFL, MDM2, TSC1	Increase; decrease 181	Viability, proliferation, invasion	18, 181, 182, 183
miR‐26a	PTEN, ATM	Increase; decrease 184	Radioresistance	184, 185
miR‐26b	EPHA2	Decrease	Proliferation, migration, invasion, vasculogenic mimicry	186
miR‐27a	FOXO3a	Increase; decrease with higher grade 187	Proliferation, apoptosis, invasion	187, 188
miR‐27b		Increase	Proliferation, apoptosis, invasion	189
miR‐28		Increase		18
miR‐29a/b	MCL1, PDPN	Increase; decrease 190	Proliferation, apoptosis, invasion	190, 191
miR‐30a	SOCS3, SEPT7	Increase	Proliferation, apoptosis, invasion, stemness	192, 193, 194
miR‐31	FIH1, RDX, TRADD	Decrease; increase 66	Proliferation, apoptosis, migration, invasion, stemness, angiogenesis, chemoresistance	66, 84, 149, 195, 196, 197
miR‐32	MDM2, TSC1	Decrease	Survival, proliferation	198
miR‐34a	MET, NOTCH1, NOTCH2, CCND1, CDK6, RICTOR, SIRT1	Decrease	Survival, proliferation, apoptosis, migration, invasion, stemness	31, 199, 200, 201, 202
miR‐92a	BCL2L11	Increase	Proliferation, apoptosis	203
miR‐92b	DKK3, NLK	Increase	Proliferation, apoptosis, invasion	204, 205
miR‐93	ITGB3	Increase	Survival, proliferation, angiogenesis, stemness	83, 148
miR‐95		Decrease		148
miR‐100	SMRT/NCOR2, ATM	Decrease; increase 206	Proliferation, apoptosis, radioresistance	206, 207
miR‐101	KLF6	Decrease	Proliferation, apoptosis, invasion, migration	149, 208
miR‐106a	SLC2A3, TIMP2	Decrease; increase in GSCs 172	Proliferation, invasion, metabolism	172, 209
miR‐107	SALL4, NOTCH2, CDK6	Decrease	Proliferation, apoptosis, invasion	210, 211, 212, 213
miR‐124	PIM3, NRAS, SOS1, PPP1R3L, RRAS, NRAS, SNAI2, MAPK14, TEAD1, SERP1, LAMB1, CDK4, IQGAP1	Decrease	Proliferation, apoptosis, migration, invasion, stemness, angiogenesis, chemoresistance, radioresistance, stress response	29, 148, 156, 214, 215, 216, 217, 218, 219, 220, 221, 222
miR‐125a	NRG1, PDPN	Decrease	Proliferation, apoptosis, invasion, migration	190, 223
miR‐125b	LIN28, BAK1, MAPK14, CDK6, CDC25A, BMF, MAZ, E2F2	Decrease^80^, ^224^, ^225^, ^226^, ^227^; increase 228, 229, 230	Survival, proliferation, apoptosis, invasion, stemness, angiogenesis, chemoresistance	80, 224, 225, 226, 227, 228, 229, 230, 231, 232
miR‐126		Decrease		233
miR‐128	P70S6K1, SUZ12, BMI1, PDGFR*α*, EGFR, E2F3a, WEE1, MSI1	Decrease	Proliferation, apoptosis, angiogenesis, stemness, radioresistance	25, 26, 27, 28, 82, 90, 148, 178, 234, 235
miR‐130a		Decrease	Chemoresistance	236, 237
miR‐130b	MST1, SAV1	Increase	Stemness	18, 238
miR‐132		Increase; decrease 148		148, 239, 240
miR‐135b	ADAM12, SMAD5, GSK3*β*	Decrease	Proliferation, migration, stemness, radioresistance	241, 242
miR‐136	AEG1, BCL2	Decrease	Apoptosis, chemoresistance	243, 244
miR‐137	RTVP1, PTGS2, MSI1	Decrease	Proliferation, apoptosis, migration, invasion, stemness	29, 245 27, 149, 246, 247, 248
miR‐139	ELTD1, MCL1	Decrease	Proliferation, apoptosis, chemoresistance	148, 249 250
miR‐140		Increase		18, 156
miR‐143	HK2, RAS	Decrease; increase 251	Migration, invasion, angiogenesis, chemoresistance, stemness, glycolysis	251, 252, 253
miR‐145	CTGF, ABCG2	Decrease; increase 251	Proliferation, migration, invasion	251, 254, 255
miR‐146	NOTCH1	Decrease; increase 84	Proliferation	84, 256
miR‐148a	FIH1	Increase	Proliferation, stemness, angiogenesis	27, 84
miR‐152	KLF4, MMP3, XIST	Decrease	Proliferation, apoptosis, migration, invasion, stemness	67, 159, 257
miR‐153	BCL2, MCL1, IRS1	Decrease	Viability, proliferation, apoptosis, stemness	258, 259, 260
miR‐155	GABRA 1, EAG1, MAPK13, MAPK14, FOXO3a, MXI1	Increase	Proliferation, apoptosis, invasion, chemoresistance	261, 262, 263 264, 265 237, 266
miR‐181	FOS, KPNA4, MGMT, RAP1B, BCL2, NOTCH2, MGMT, KRAS, BCL2, MDM2	Decrease	Proliferation, apoptosis, invasion, stemness, chemoresistance, radioresistance	88, 111, 178, 234, 267, 268, 269, 270, 271, 272, 273, 274, 275
miR‐182	BCL2L12, HIF2A, MET, CYLD, LRRC4	Increase	Proliferation, apoptosis, invasion, angiogenesis, chemoresistance, stemness	19, 135, 276, 277
miR‐184	FOXO3, SND1	Decrease	Proliferation, invasion, chemoresistance	18, 278 279
miR‐193	SMAD3	Increase	Proliferation	84, 280
miR‐195	CCND1, CCNE1	Decrease; increase in TMZ‐resistant GBM 87	Proliferation, chemoresistance	281 178, 87
miR‐196	NFKBIA	Increase	Proliferation, apoptosis	85, 178, 248, 282, 283
miR‐200a/b	MGMT	Decrease; increase	Chemoresistance	84, 284
miR‐203	SNAI2, PLD2	Decrease	Chemoresistance	285, 286, 287
miR‐205	VEGFA, LRP1	Decrease	Proliferation, apoptosis, invasion, migration	102, 288, 289
miR‐210	HIF3A	Increase	Survival, chemoresistance	18, 81, 237
miR‐218	LEF1, IKBKB, BIM1, ECOP, CDK6	Decrease	Survival, proliferation, apoptosis, migration, invasion, stemness	54, 55, 290, 291, 292, 293
miR‐221/222	PTEN, PUMA, CDKN1B, CDKN1C, PTPRM, MGMT, SEMA3B, TIMP3, GJA1	Increase; decrease 84, 178	Viability, proliferation, apoptosis, migration, invasion, chemoresistance, radioresistance	234, 294 48, 149, 178 52, 84, 295, 296, 297, 298
miR‐296‐3p	EAG1	Decrease	Proliferation, chemoresistance	299
miR‐302/367 cluster	CXCR4		Invasion, chemoresistance, stemness	300, 301
miR‐320	E2F1	Decrease	Proliferation, migration	302
miR‐323	IGFR1	Decrease; increase 237	Proliferation, apoptosis, migration	237, 303
miR‐326	NOB1	Decrease	Proliferation, apoptosis	304, 305 237
miR‐328	SRFP1	Decrease; increased in low grade GBM 306	Proliferation, invasion	18, 96, 306
miR‐329	E2F1	Decrease; increase 237	Survival, proliferation, apoptosis	237, 307
miR‐330	SH3GL2	Increase; decrease 149	Proliferation, apoptosis, migration, invasion	149, 308, 309
miR‐331‐3p	NRP2, HER2	Decrease	Proliferation, apoptosis, migration, invasion, angiogenesis	145, 310
miR‐335	DAAM1, PAX6	Increase	Survival, proliferation, apoptosis, invasion, stemness	53, 311, 312, 313
miR‐340	PLAT, ROCK1, CDK6, CCND1, CCND2	Decrease	Proliferation, apoptosis, migration, invasion, stemness	314, 315, 316
miR‐363	BIM, CASP3	Increase	Survival	317
miR‐372	PHLPP2	Increase	Proliferation, apoptosis invasion	318
miR‐377	SP1	Decrease	Proliferation, invasion	319
miR‐378		Decrease	Migration, invasion	320
miR‐381	LLRC4	Increase	Proliferation	277, 321
miR‐410	MET	Decrease	Proliferation, invasion	322
miR‐451	CAB39	Decrease	Proliferation, invasion, apoptosis, stemness	56, 323, 324
miR‐455‐3p	SMAD2	Increase	Chemoresistance	87, 325
miR‐483‐5p	ERK1	Decrease	Proliferation	326
miR‐487b		Decrease	Proliferation, apoptosis	148
miR‐491‐3p	IGFBP2, CDK6	Decrease	Proliferation, invasion, stemness	327
miR‐491‐5p	BCL2L1, EGFR, CDK6, MMP9	Decrease	Proliferation, invasion, stemness	327, 328
miR‐513	LRP6	Decrease	Proliferation	329
miR‐582‐5p	CASP3, CASP9	Increase	Proliferation, apoptosis	317
miR‐603	MGMT, WIF1, CTNNBIP1	Increase	Proliferation, chemoresistance	268, 330
miR‐655	SENP6	Increase	Invasion	331
miR‐663	PIK3CD	Decrease	Proliferation, invasion	332
miR‐873	IGF2BP1, BCL2	Decrease; increase 260	Proliferation, apoptosis, migration, invasion, chemoresistance	148, 333, 334
miR‐874		Decrease		156

ECOP, epidermal growth factor receptor coamplified and overexpressed protein; KLF4, Krüppel‐like factor 4; MAZ, myc‐associated zinc finger protein; MMP, matrix metalloproteinase; PUMA, p53‐upregulated modulator of apoptosis;TMZ, temozolomide.

### Enhancing cell proliferation

The ability to continuously proliferate is a fundamental characteristic of all cancers that is achieved through deregulation of cell signaling pathways. Importantly, miRNAs can influence the promotion of sustained signaling for proliferation as well as the ability to evade growth suppressors, enhancing the expansion capacities of cancer cells.

This is exemplified by survival and proliferation pathways modulated by EGFR and Akt. In GBM, where amplification of *EGFR* is a characteristic trait of primary tumors, miRNAs involved in *EGFR* regulation display corresponding disruptions in expression with disease progression. miR‐7, which acts as a tumor suppressor, directly targets *EGFR* and can independently repress the Akt pathway through targeting of its upstream regulators. In GBM, miR‐7 is frequently downregulated, allowing for enhanced activation of the Akt pathway, and thus increased viability and invasiveness of tumor cells 23, 24. These protumorigenic effects can be corrected with transfection of mimic miR‐7 oligonucleotides, which results in decreased invasiveness and increased apoptosis of GBM cell lines 23, 24.

miR‐128 also exerts an antiproliferative effect and is frequently downregulated with GBM 25. Its growth‐suppressive functions are mediated through numerous pathways via targeting of (1) *EGFR* and growth factor receptor *PDGFRA* 25, (2) *WEE1,* a regulator of cell cycle progression that can block entry into mitosis through inhibition of CDK1 26, (3) *MSI1*, which regulates the expression of the NOTCH1 antagonist *NUMB*, influencing proliferation and the maintenance of stem cells 27, and (4) the transcription factor *E2F3A*, which is a cell cycle activator 28. Thus, repression of miR‐128 in cancer cells can be an advantageous mechanism for overcoming normal proliferative signaling.

However, these pathways can also be targeted by other miRNAs. The E2F transcription factor family plays a pivotal role in the cell cycle. In response to mitogenic signaling, RB1 is phosphorylated by CDK/cyclin complexes leading to activation of E2F‐responsive genes, thereby promoting cell cycle progression. This pathway can be suppressed by miR‐137 and miR‐124a, which inhibit *CDK6* expression as well as phosphorylation of RB1. As both miR‐137 and miR‐124a are consistently downregulated in GBM 29, transfection of either miRNA can prevent proliferation of GBM cells. Further, forced miR‐137 overexpression can also mediate *MSI1* 27 and *PTGS2* 30, which additionally function to reduce proliferation and invasion.

miR‐34a is also persistently downregulated in GBM and has similar targets, repressing *CDK6*,* CCND1*,* MET, NOTCH1, NOTCH2*, and *SIRT1* 31, 32, 33, 34. Of particular importance to proliferative signaling, targeting of *CDK6* and *CCND1* prevents the downstream prosurvival signaling of the cyclin/CDK pathway. Thus, restoration of miR‐34a can reduce CDK6 protein expression, inhibiting cell survival, proliferation, and invasion as well as inducing apoptosis 31, 32.

RAS proteins, which process signals downstream of growth receptors, are also targeted by miRNAs and play a key role in the deregulation of proliferation pathways in many cancers, including GBM. let‐7 is an miRNA which is decreased in gliomas and inversely correlates with the presence of RAS proteins. Restoration of let‐7 reduces the expression of RAS, resulting in decreased proliferation and migration of tumor cells in vitro and inhibition of tumor growth in vivo. Importantly, transfection of let‐7 has no effect on normal human astrocytes, indicating that normal cells are able to more effectively regulate miRNA activity 35.

In addition to miRNA modulation of RAS proteins, RAS proteins can conversely influence miRNA expression. Indeed, miR‐21 is a transcriptional target of the AP‐1 complex and RAS oncogenes are well‐known inducers of AP‐1 activity. AP‐1 is a transcription factor that regulates a variety of target genes, leading to an increase in cell proliferation, invasion, and angiogenesis during tumor development. AP‐1 induces miR‐21, which downregulates tumor suppressors PDCD4 and PTEN. Inhibition of PDCD4 then contributes to an increase in AP‐1 activity, revealing an AP‐1 autoregulatory mechanism in RAS transformation 36. In addition to PDCD4 and PTEN, miR‐21 also exerts its prooncogenic effect by downregulating numerous targets including *ANP32A, SMARCA4, SPRY2, IGFBP3, LRRFIP1,* and *RECK*. Through these targets, miR‐21 can influence numerous biological processes in addition to the promotion of cell cycle progression, including promotion of invasion and metastasis and resistance to chemotherapeutics 37, 38, 39, 40, 41, 42, 43, 44. Notably, inhibition of miR‐21 expression can repress tumor growth 37, 43, 45.

### Resisting cell death

In addition to sustained proliferation, the ability to evade apoptosis is an essential characteristic of cancer. Dysregulation of miRNA expression is one mechanism for allowing cancer cells to bypass signals for programmed cell death. Further, miRNA mediation of apoptosis is strongly linked to drug resistance, as many treatments aim to initiate apoptotic pathways by damaging cancer cells. Thus, the ability to override signals for cell death is critical for not only sustained proliferation but also for acquiring drug immunity.

miRNAs can have either pro‐ or antiapoptotic functions and are therefore differentially regulated during cancer progression. Antiapoptotic miRNAs target proapoptotic genes and are frequently upregulated with GBM. miR‐21 is antiapoptotic and enhances tumor formation by targeting the signaling pathways of P53 and TGF‐*β* as well as the mitochondrial apoptotic pathway 46. Inhibition of miR‐21 results in activation of caspases, suspension of cell growth, reduced invasion, increased apoptosis, and enhanced chemosensitivity. These effects are mediated in part by decreased repression of targets including *HNRPK, TAP63*, and *PDCD4* 37, 44, 46. Further, miR‐21 can modulate the extrinsic apoptotic pathway through downregulation of *FASL*, an ability that has been particularly evident in cancer stem cells 47. Thus, miR‐21 has widespread effects on cell death pathways, making it a critical player in GBM pathogenesis and a promising target for therapeutic interventions.

miR‐221 and miR‐222 are also overexpressed in GBM and have numerous targets involved in gliomagenesis, including in apoptotic pathways. miR‐221/222 can regulate cell death through targeting of p53‐upregulated modulator of apoptosis (PUMA). Under normal conditions, PUMA binds Bcl‐2 and Bcl‐x, rapidly inducing apoptosis. Thus, forced expression of miR‐221/222 and the subsequent downregulation of *PUMA,* promotes cell survival. Therefore, the knockdown of miR‐221/222 is able to induce cell death and decrease tumor growth 48, 49. miR‐221/222 can also target the cell growth‐suppressive CDK inhibitors P27 and P57 50 and thus are tightly linked to cell cycle checkpoints for initiation of S phase. When human glioblastoma U251 cells are treated with antisense miR‐221/222, the cell cycle is arrested in G0 or G1 phase 51. Moreover, treatment with antisense oligonucleotides for miR‐221/222 enhances the effects of both temozolomide (TMZ) and radiation 49, 52.

miR‐335 is also antiapoptotic and is upregulated with GBM. miR‐335 targets potential tumor suppressor, disheveled‐associated activator of morphogenesis 1 (DAAM1), thereby promoting growth and invasion of astrocytoma cells. Thus, inhibition of miR‐335 is able to effectively suppress growth and induce apoptosis of astrocytoma cells both in vitro and in vivo. Indeed, delivery of a miR‐335 antagonist to rat glioma C6 cells resulted in growth arrest, activation of apoptosis, repression of invasion, and marked regression of astrocytoma xenografts 53. Contributing to its widespread influence on cellular function, miR‐335 also regulates *RB1* and controls cell proliferation in a p53‐dependent manner 53.

On the opposing side of the spectrum are proapoptotic miRNAs, which target antiapoptotic genes and are often downregulated during GBM progression. miR‐218 is proapoptotic and exerts its influence through targeting of *CDK6*. Thus, its downregulation corresponds with an increase in cellular proliferation with GBM. Exogenous administration of miR‐218 can effectively repress expression of *CDK6*, inhibiting cell proliferation and inducing apoptosis of malignant glioma cells 54. Further, miR‐218 was found to sensitize glioma cells to apoptosis by regulating epidermal growth factor receptor coamplified and overexpressed protein (ECOP), which can suppress the transcriptional activity of NFκB, thereby modulating the cellular apoptotic response 55.

miR‐451 also acts as a tumor suppressor and is subject to downregulation in GBM tissue. Correcting the loss of miR‐451 via administration of miR‐451 mimics has been shown to inhibit cell growth, decrease invasive capacity, induce G0/G1 phase arrest, and increase apoptosis of GBM cells. This activity is predicted to result from modulation of the PI3K/Akt pathway as administration of miR‐451 mimics results in decreased protein expression of Akt1, Cyclin D1, MMP‐2, MMP‐9, and Bcl‐2 as well as increased expression of p27 56.

#### Autophagy

An additional means of miRNA regulation of cell death is through modulation of autophagy, an evolutionarily conserved, multistep lysosomal degradation process that functions to catabolize unnecessary or dysfunctional cellular components. It is unclear whether autophagic activity in dying cells is the cause of death or a survival mechanism, and thus, the role of autophagy in cancer has not been completely elucidated. Autophagy may protect against cancer by isolating damaged organelles, promoting cell differentiation, increasing protein catabolism, or potentially by prompting apoptosis of cancerous cells. Alternatively, autophagy can contribute to cancer progression by degrading apoptotic mediators and by providing a means of surviving nutrient depletion or absence of growth factors 57.

Various miRNAs contribute to both autophagy and progression of GBM. miR‐17 is frequently overexpressed in gliomas and targets *ATG7,* one of the master regulators of autophagy. Downregulation of miR‐17 promotes ATG7 protein expression and induction of autophagy, leading to a subsequent decrease in cell viability and proliferation. Moreover, anti‐miR‐17 treatment can enhance the effectiveness of radiation and TMZ, indicating that modulation of autophagy is an important aspect of GBM survival and progression 58.

miR‐21 also inhibits autophagy and enhances resistance of glioma cells to radiation therapy 59. A study by Gwak et al. 59 found that miR‐21 expression levels positively correlated with resistance to radiation in glioma cells. Anti‐miR‐21 treatment of U373 and U87 malignant glioma cell lines resulted in an increased sensitivity to radiation. Further, administration of anti‐miR‐21 was correlated with an increased expression of molecular factors associated with autophagosome formation as well as autophagic activity. This increase in autophagy resulted in an expansion of the apoptotic population following irradiation 59. Thus, these studies indicate that autophagy may play a protective role against cancer, which could potentially be exploited using miRNA‐based therapies.

#### Enabling replicative immortality

While normal cells have a limited number of potential divisions, cancer cells frequently display infinite replicative capacity. This is particularly evident in cancer stem cells, which serve as potent drivers of tumor growth and development. Indeed, CD133^+^ GBM cancer stem cells (GSCs) are able to initiate tumors with as few as 100 cells when injected into the brains of NOD‐SCID mice 60. Due to their unique characteristics and extremely aggressive nature, including resistance to radio‐ and chemotherapy 61, it has been proposed that GBM cells, and GSCs in particular, may originate from adult neural stem cells or neural precursors that have undergone transformation, allowing them to retain stem cell characteristics 62.

In support of stem cell origins, Lavon et al. 63 demonstrated that gliomas display neural precursor cell (NPC)‐like miRNA signatures. In a human glioma panel, 71 of the 180 miRNAs investigated had a distinct expression pattern which matched that of the embryonic stem cells and neural precursor cells. This included upregulation of miR‐21 and downregulation of miR‐124 and miR‐137, which have previously been implicated in gliomagenesis. Interestingly, about half of the miRNAs expressed in the shared profile clustered in seven genomic regions which are prone to genetic or epigenetic aberrations with various cancers. Further, this expression signature was consistent across cell lines as well as unsorted GBM human tumors, indicating that a majority of GBM tumor cells may share this profile.

Silber et al. 29 likewise confirmed that miR‐124 and miR‐137 were significantly decreased in GBM as compared to normal brain tissue. They further found that miR‐124 and miR‐137 induced differentiation of adult mouse neural stem cells and human GBM‐derived stem cells, suggesting that repression of these miRNAs is indeed critical for maintenance of stem cell properties.

miRNAs have also been associated with various facets of the selection and promotion process for GSCs. For example, miR‐128 targets the oncogene *BMI1* and is downregulated in GBM, allowing for enhanced self‐renewal of GSCs 64. miR‐326 is also decreased in gliomas and its expression is inversely correlated with expression of smoothened (SMO), a component of the Hedgehog pathway. The Hedgehog pathway is a signaling cascade that is important for the modulation of embryonic stem cells as well as for the proliferation of adult stems cells. Overexpression of miR‐326 was able to repress self‐renewal and partially prompted differentiation of U251 tumor stem cells. Further, the transfection of miR‐326 reduced intracranial tumorigenicity 65.

A study by Wong et al. 66 found that miRNAs can also regulate GBM growth through maintenance of tumor stem cells and stem cell niches. Inhibition of miR‐148a and miR‐31 using antisense oligonucleotides was able to reduce cancer cell proliferation, deplete stem cells, and normalize tumor vasculature. These effects were mediated in part through miR‐148a and miR‐31 repression of factor‐inhibiting hypoxia‐inducible factor 1 (FIH1), which can influence angiogenesis and tumor stemness through HIF1*α* and Notch signaling 66. Thus, their inhibition was able to effectively suppress tumor growth and prolong survival time in orthotopic xenograft GBM mouse models.

Other miRNAs, such as miR‐152, serve as tumor suppressors and are downregulated in GSCs. Restoration of miR‐152 is able to reduce proliferation, migration, and invasion, as well as induce apoptosis of GSCs. These activities are mediated through targeting of Krüppel‐like factor 4 (KLF4), which is associated with downregulation of LGALS3 and reduced activation of the MEK1/2 and PI3K signaling pathways. Importantly, miR‐152 overexpression is able to decrease tumor volume and enhance survival time in mouse models 67.

### Activating invasion and metastasis

While metastasis to other parts of the body is rare, GBM is characterized by extensive, diffuse infiltration throughout the brain. This capacity for pervasive invasion and metastasis is enabled through various mechanisms including modulation of cell‐to‐cell and cell‐to‐matrix interactions, degradation and remodeling of the extracellular matrix, cytoskeletal reorganization, and gain of migratory behavior. Epithelial to mesenchymal transition (EMT) is an important part of this process. EMT is characterized by the loss of an epithelial phenotype, including expression of E‐cadherin, and the acquisition of mesenchymal markers such as fibronectin, vimentin, and N‐cadherin. Many distinct molecular processes occur during EMT including activation of transcription factors, expression of specific cell‐surface proteins, reorganization of cytoskeletal proteins, and production of extracellular matrix‐degrading enzymes 68. As a result, tumor cells develop increasing invasive and migratory potential. Importantly, these processes are accompanied by changes in expression of many miRNAs, which could serve as potential targets for inhibiting invasion and metastasis.

As previously noted, miR‐21 is frequently overexpressed during gliomagenesis and contributes to a variety of protumorigenic pathways. Through the targeting of matrix metalloproteinase (MMP) inhibitors, such as *RECK* and *TIMP3*, as well as tumor suppressors including *ANP32A* and *SPRY2*, miR‐21 can increase the expression and activity of various MMPs, facilitate Ras/Raf binding, and induce ERK phosphorylation, thereby enhancing the invasive potential of GBM cells 40, 69, 70. Administration of antisense oligonucleotides to miR‐21 is able to ameliorate these effects, resulting in elevated levels of *RECK* and *TIMP3*, a reduction in MMP activity, and decreased migration and invasion of GBM cells 70.

MMPs are also targeted by other miRNAs, including miR‐146b. miR‐146b inhibits expression of MMP16, an enzyme that functions in proteolysis of extracellular matrix components and is therefore critical for the migration and invasion properties of tumor cells. Thus, miR‐146b is often decreased during gliomagenesis, allowing for upregulation of *MMP16* 71.

miR‐10b can also modulate MMPs, indirectly targeting MMP14 as well as urokinase receptor (uPAR) and RhoC through direct suppression of their upstream target *HOXD10*. Augmented miR‐10b expression levels are correlated with higher grade gliomas, 72, 73 and indeed, GBM cells display reduced growth, invasion, and angiogenesis, as well as enhanced cell death when treated with antisense oligonucleotides to miR‐10b 73. Further, in an orthotopic human glioma mouse model, inhibition of miR‐10b diminished the growth, invasiveness, and angiogenicity of glioma cells in the brain, significantly prolonging the survival of glioma‐bearing mice 74.

miR‐7 also has many targets involved in metastasis including *FAK, PI3K, EGFR,* and *RAF1* 23, 24, 75, 76. As indicated by its targets, miR‐7 functions as a tumor suppressor and is downregulated in GBM. Its overexpression can inhibit metastasis and invasion of GBM cells by directly repressing *FAK*, a mediator of cell‐extracellular matrix signaling, as well as by reducing the expression of *MMP2* and *MMP9*, thereby decreasing the ability of GBM cells to move through extracellular matrix 75. Further, by suppressing *EGFR* expression and inhibiting the Akt pathway, miR‐7 can decrease the viability and invasiveness of GBM cells 23.

### Inducing angiogenesis

GBM is distinguished from lower grade gliomas by excessive microvascular proliferation, which results in highly vascularized tumors. Enhanced vascularization allows for increased proliferative and invasive capacity of cancer cells due to the greater availability of oxygen and nutrients. Additionally, blood vessels can aid in cell migration, supporting diffuse infiltrative migration of single cells into the brain parenchyma as well as perivascular migration of cells along the microvasculature 77.

A group of miRNAs now termed angiomiRs, have recently been identified as important contributors to neovascularization in GBM 78. These molecules can act on either tumor cells or neighboring tumor‐associated cells. miR‐296 is an angiomiR that has been found to be upregulated in endothelial cells with the presence of glioma cells or angiogenic growth factors such as VEGF. Augmented expression of miR‐296 is associated with increased endothelial cell tube formation and enhanced vascularization of tumors, while knockdown of miR‐296 results in reduced tumor angiogenesis 79.

miR‐125b is an angiomiR that has conversely been found to be repressed in GBM‐associated endothelial cells. This downregulation results in increased expression of its target, myc‐associated zinc finger protein (MAZ), a transcription factor that regulates VEGF. Decreased expression of miR‐125b in target cells promotes vascularization of tumors 80.

Within GBM tumor cells, hypoxia can also influence miRNA expression as a mechanism for augmenting angiogenesis. Exemplifying this, hypoxia induces expression of miR‐210‐3p, which directly targets *HIF3A*, a negative regulator of hypoxic response that acts through downregulation of VEGF. Thus, miR‐210‐3p overexpression induces HIF, VEGF, and CA9 transcriptional activity, enhancing vasculogenesis. However, inhibition of miR‐210‐3p under hypoxic conditions prevents HIF‐mediated induction of VEGF and CA9, reducing vascular density, and abating growth of tumors in vivo 81.

miR‐128 is also able to modulate angiogenesis, functioning through suppression of *P70S6K1*, a kinase upstream of HIF‐1*α* and VEGF. miR‐128 is decreased in gliomas, which promotes angiogenesis, cell proliferation, and tumor growth. Restoration of miR‐128 is able to attenuate these effects, while forced expression of *P70S6K1* can partly rescue the inhibitory function of miR‐128 on cancer growth 82.

miR‐93, a member of the miR‐17 family and part of the miR‐106b‐25 cluster, is upregulated in GBM and enhances cell survival, sphere formation, and tumor growth, in part through promotion of angiogenesis. miR‐93‐expressing cells induce blood vessel formation, potentially through suppression of integrin‐*β*8, a protein involved in cell–cell and cell–matrix interactions. Fang et al. 83 found that vasculogenesis could be enhanced by overexpressing miR‐93 in the human glioblastoma cell line U87 and then coculturing the GBM cells with endothelial cells. This resulted in an increase in endothelial cell proliferation and tube formation in vitro and highly increased blood vessel formation in GBM xenograft tumors in mice in vivo 83. These studies illustrate the important role that miRNAs play in the cross‐talk between cells, serving as critical mediators in tumor cell modulation of their microenvironment.

### miRNAs in GBM drug resistance

Currently, the standard treatment for newly diagnosed GBM is cytoreductive surgery followed by concurrent radiation and chemotherapy with TMZ 2. However, even the most successful treatment plans are only palliative, aiming to delay relapse for as long as possible. A major challenge is that the response to treatments is highly variable across patients. This is largely due to the heterogeneity of tumors, including differences in genotypes and miRNA profiles between each tumor. However, an advantage is that the correlation between miRNA expression levels and GBM progression can be exploited and miRNAs can be used as predictors of treatment response or overall survival. Indeed, Srinivasan et al. 84 found a 10‐miRNA signature of GBM tumors that was associated with patient survival. The signature included three miRNAs (miR‐20a, miR‐106a, and miR‐17‐5p) that were protective and seven (miR‐31, miR‐222, miR‐148a, miR‐221, miR‐146b, miR‐200b, and miR‐193a) that were risky with respect to the association between their expression and patient survival. Additionally, other studies have found that high expression of miR‐21, miR‐182, and miR‐196 as well as low expression of miR‐181b and miR‐106a are associated with poor patient outcomes 19, 85, 86.

As the standard GBM treatment utilizes radiation and TMZ, many studies have focused on the potential role of miRNAs in resistance to these therapeutics. To identify miRNAs involved in TMZ resistance in GBM, Ujifuku et al. 87 used miRNA microarrays to perform a comprehensive analysis of miRNA expression in a TMZ‐sensitive GBM cell line. They found that miR‐195, miR‐455‐3p, and miR‐10a^∗^ were the three most upregulated miRNAs in resistant cells. Of the three, knockdown of miR‐195 had the greatest effect on initiating tumor cell death, significantly enhancing the effectiveness of TMZ. Slaby et al. 88 also examined the correlation between expression levels of selected miRNAs and TMZ resistance in GBM, but used 22 primary GBM tumors instead of cell lines. They found that miR‐221, miR‐222, miR‐181b, miR‐181c, and miR‐128 were significantly downregulated with GBM, while miR‐21 was overexpressed. Downregulation of miR‐181b and miR‐181c had the strongest correlation with responsiveness to TMZ treatment, indicating that their presence could serve as a predictive marker for response to TMZ therapy.

As miR‐21 is consistently upregulated in GBM and targets numerous pathways including those involved in survival, proliferation, invasion, and apoptosis, it is unsurprising that it has also been found to play a role in drug resistance. Shi et al. 89 determined that overexpression of miR‐21 significantly inhibited the effect of TMZ on apoptosis, which was mediated through downregulation of proapoptotic proteins Bax and caspase‐3 as well as upregulation of antiapoptotic protein Bcl‐2. Moreover, numerous other studies have investigated the impact of miR‐21 on drug resistance in GBM, finding that inhibiting miR‐21 can enhance the chemosensitivity of human GBM cells to TMZ, paclitaxel, sunitinib, doxorubicin, and VM‐26 39, 90, 91, 92, 93, 94.

miRNAs can also influence drug resistance by targeting the multidrug resistance protein ABCG2 (ATP‐binding cassette subfamily G member 2), a transporter which regulates shuttling of substrates across the cellular membrane. miR‐328 directly targets ABCG2 and is underexpressed in many cancers, including GBM. This allows for elevated expression of ABCG2, enhancing chemoresistance 95. Moreover, high expression of miR‐328 is a protective factor in GBM, while low levels are associated with shorter survival times 96. Thus, restoration of miR‐328 may be an effective option for combination therapy with radiation or chemotherapeutics.

### Extracellular miRNAs and GBM

miRNAs are present and stable at high levels in body fluid, including serum, plasma, saliva, urine, and milk. Additionally, miRNA profiles and concentrations in body fluids are correlated with pathological conditions such as cancer, suggesting that miRNAs could serve as promising noninvasive prognostic and diagnostic biomarkers 97, 98. Thus, in tumors such as GBM, where it is often difficult to obtain tissue samples, miRNA profiling via liquid biopsies could provide a promising alternative.

Wang et al. 99 characterized plasma‐derived miRNA expression profiles, finding that with GBM, miR‐21 was upregulated while miR‐128 and miR‐342‐3p were downregulated. Further, expression levels correlated with glioma stage and could be used to distinguish gliomas from other brain tumors such as pituitary adenomas and meningiomas. Roth et al. 100 also explored the blood‐derived miRNA profiles of GBM patients, substantiating a decrease in miR‐342‐3p, but conversely finding that expression of miR‐128 was significantly increased with disease. However, a more recent study investigating miR‐128 supported Wang et al., finding that it was significantly decreased in the serum of glioma patients prior to surgery, and was elevated post surgery (although still not reaching normal levels) 101.

miR‐205 has also been identified as a potential biomarker due to its significantly lower levels in the serum of GBM patients as compared to controls. In a study of 64 glioma patients, serum miR‐205 levels displayed a stepwise decrease with ascending tumor grade and additionally increased following surgery and decreased again during tumor recurrence. Further, patients with advanced pathological grade demonstrated longer overall survival times when serum miR‐205 levels were elevated 101, 102. Thus, serum miRNAs could serve as critical indicators of disease progression and outcome.

The mechanisms for release of miRNAs from cancer cells as well as for crossing the blood–brain barrier to enter systemic circulation are not completely understood. Potential means of release and stabilization include binding of miRNAs to protein complexes or packaging within extracellular vesicles. Studies investigating the presence of extracellular miRNAs have found that a vast majority of those present in blood or cell culture media are independent of exosomes and are bound to Ago2, which enhances their stability 103, 104. However, the selection mechanism for release of miRNAs into extracellular space has yet to be elucidated. Turchinovich et al. 103 proposed that instead of being actively released, large portions of circulating miRNAs could be derived from dead or dying cells.

In addition to protein attachment, horizontal transfer of miRNAs from cancer cells could be facilitated by extracellular vesicles (EV), which are able to protect proteins and nucleotides from degradation 105. Although it has been suggested that a minority of miRNAs in circulation are found in EVs, these vesicles may represent a potent means of paracrine signaling to nearby cells. Cancer cells are able to use EVs to export selected molecules as a mechanism for modulating the tumor microenvironment and establishing premetastatic niches, thereby facilitating dissemination and distant engraftment. The extracellular presence of miRNAs suggests that they may be involved in this process, contributing to the metastatic potential of cancer cells and the mediation of communication between cancer cells and their environment.

Extracellular vesicles such as exosomes and microvesicles, are among the most well‐studied mechanisms of lateral transfer. While microvesicles bud directly from the plasma membrane, exosomes are formed from intraluminal vesicles, which bud into early endosomes and then form multivesicular endosomes (MVE). MVEs are then released by either fusion with the plasma membrane or direct release from the plasma membrane 106. Importantly, the release of miRNAs via exosomes is selective, based on factors such as cell of origin and malignancy. For example, while EVs derived from dendritic cells contain costimulatory proteins necessary for T‐cell activation, the content of vesicles from tumor cells more frequently aid in tumor growth and invasiveness 107. Additionally, Pigati et al. 108 found that approximately 30% of the miRNAs released into exosomes did not reflect the intracellular profile. In particular, a majority of the miR‐451 and miR‐1246 produced by malignant mammary epithelial cells was released, while the majority of these same miRNAs produced by nonmalignant mammary epithelial cells were retained, indicating that there is a selection mechanism for miRNA release 108. Moreover, selectively exported miRNAs from malignant cells may be packaged in structures that differ from normal cells and further, some miRNAs exclusively associate with certain vesicles 109.

The formation of EVs to transport cellular information has been confirmed in GBM. Cultured primary cells from GBM tumors were found to be covered with microvesicles of various sizes, containing both RNA and proteins 107. Moreover, the exosomes from GBM tumors were internalized by endosome‐like structures of brain endothelial cells, where they were able to stimulate tubule formation. This confirmed that exosomes can serve as a means of intercellular communication and thus are a critical tool for tumor cells to use in influencing proximal cells 107. GBM microvesicles were also able to stimulate the proliferation of human glioma cell lines, indicating a potential self‐promoting function 107.

miRNAs released from tumor cells have also been shown to modulate the tumor microenvironment. Tominaga et al. 110 discovered that miR‐181c was significantly upregulated in EVs from breast cancer cells, particularly in brain metastatic breast cancer. Further, miR‐181c was able to trigger the breakdown of an in vitro model of the blood–brain barrier. EVs from the cancer cells were selectively incorporated into endothelial cells (although not into pericytes or astrocytes), where miR‐181c suppressed *PDPK1*, initiating downregulation of phosphorylated cofilin. This led to abnormal localization of actin and thus weakening of the blood–brain barrier 110. However, miR‐181c has also been shown to act as a tumor suppressor, undergoing downregulation in GBM 111. This suggests that miRNAs may have different functions depending on the cancer type and microenvironment.

An additional example of miRNA regulation of the tumor microenvironment is demonstrated by miR‐105. miR‐105 released from cancer cells is able to destroy tight junctions through inhibition of tight junction protein ZO‐1. Downregulation or loss of tight junctions contributes to cancer progression by altering cell migration, proliferation, polarity, and differentiation. Further, overexpression of miR‐105 in nonmetastatic cancer cells induces metastasis as well as vascular permeability in distant organs. Fittingly, miR‐105 can be detected in circulation at the premetastatic stage. Moreover, its levels in the blood and tumor are associated with ZO‐1 expression and metastatic progression in early‐stage breast cancer 112.

It is important to note that exosomal transfer of miRNAs is not exclusively involved in cancer promotion and can also serve as a physiological mechanism for suppressing cancer proliferation. Kosaka et al. 113 demonstrated that noncancerous cells can secrete and transfer antiproliferative miR‐143 exclusively to cancer cells where miR‐143 is deregulated, thereby suppressing their proliferation. As cells with normal levels of miR‐143 were not affected by its overexpression, it appears that there may be a threshold for miRNA activity or that normal cells are more effectively able to regulate miRNAs.

### miRNA‐based therapeutics

Increasing evidence demonstrates that aberrant miRNA expression profiles and signaling pathways are present in many cancers. As these molecules have the ability to target several genes within the same pathway or even multiple oncogenic pathways, miRNAs represent promising therapeutic targets with potential for more comprehensive benefits than other targets with more limited activities. Importantly, miRNAs can function as either oncogenes or tumor suppressors, which then correspond to two different approaches to miRNA‐targeted therapy. These methods aim to either (1) reduce the expression of target miRNAs via antisense technologies or miRNA sponges or (2) restore miRNA expression using synthetic mimics or gene replacement therapy. There are currently a multitude of different strategies being investigated under each category.

#### Antisense oligonucleotides

**Figure 2:**
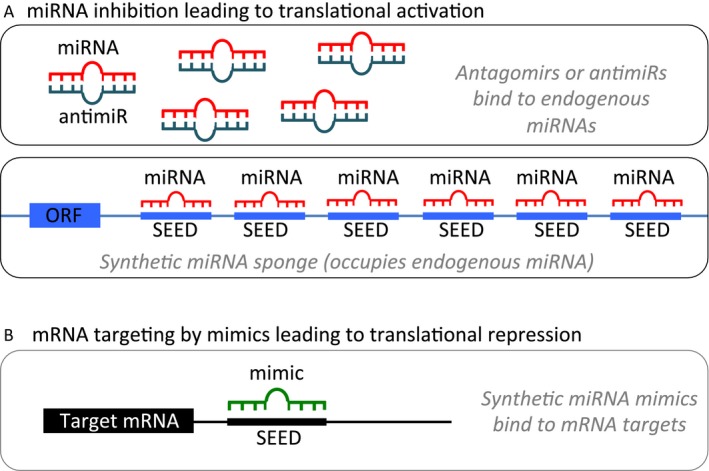
miRNA modulation strategies for therapeutic intervention. (A). miRNA inhibition. (1) Antagomirs are synthetic, single‐stranded RNA‐based oligonucleotides that are complementary to mature endogenous miRNAs, allowing for binding and silencing of their targets. (2) miRNA sponges contain multiple binding sites to an miRNA of interest, competitively inhibiting it from binding to its target mRNA. As the binding sites are specific to an miRNA's seed region, sponges can inhibit an entire family of related miRNAs. (B) miRNA mimics are synthetic, double‐stranded RNA molecules that have identical sequences to their naturally occurring equivalents, allowing for restoration or amplification of the activity of a target miRNA.

Overexpressed intracellular miRNAs can be inhibited via administration of synthetic antisense, single‐stranded RNA‐based oligonucleotides, termed antagomirs or antimiRs. Antagomirs are complementary to mature endogenous miRNAs, allowing for binding and silencing of their targets (Fig. 2A). Efficient inhibition by antagomirs requires optimization of the oligonucleotides for high binding affinity, high resistance to nuclease degradation, low toxicity, and efficient in vivo delivery. These factors can be modified through alterations of the sugar, the nucleobase, the internucleotide linkages, or with the addition of nonnucleotide modifiers 114.

Phosphorothioate oligodeoxynucleotides were the most common type of first‐generation antisense oligonucleotides, having high resistance to nucleases but low binding stability and high toxicity. These were followed by 2′‐*O*‐methyl‐modified oligonucleotides, which displayed increased binding stability and reduced nonspecific effects, but suboptimal efficiency 115. 2′‐*O*‐methyl‐modified oligonucleotides were further improved on by the development of locked nucleic acid (LNA)‐modified oligonucleotides, which have higher stability, efficacy, and specificity as well as lower toxicity 116, 117.

LNAs are modified, conformationally locked nucleotide analogs with a high level of affinity to DNA and RNA nucleotides and high nuclease resistance 118. LNA‐linked oligonucleotides are currently the most widely used class of antagomirs and have shown promise for treating various conditions. Indeed, a PBS‐formulated LNA antagomir was found to be effective at reducing serum cholesterol levels in African green monkeys. As miR‐122 plays a critical role in fat and lipid metabolism, the study utilized an LNA‐antagomir against liver‐expressed miR‐122. Upon administration, the antagomir was taken into the cytoplasm of hepatocytes, where it was able to successfully deplete miR‐122. This was accompanied by effective lowering of plasma cholesterol levels and no evidence of toxicity or histopathological changes 119.

LNA‐based antagomirs have also been utilized in studies targeting hepatitis C virus (HCV), which requires miR‐122 for replication. Treatment with LNA‐based anti‐miR‐122 oligonucleotides was able to suppress viremia and improve HCV‐induced liver pathology in chimpanzees, also without negative side effects 120. Subsequent studies resulted in the development of miravirsen, an LNA‐modified antisense oligonucleotide against miR‐122, which was the first miRNA‐targeting drug in clinical use. Phase I and II clinical trials evaluated the safety and efficacy of miravirsen, finding that patients had a dose‐dependent reduction in HCV levels and no adverse side effects 121. However, as miRNAs have widespread impact and can function in various pathways, the long‐term effects of antagomirs must be more thoroughly investigated. Notably, in other contexts, miR‐122 has been shown to function as a tumor suppressor, including in GBM, where decreased expression of miR‐122 is associated with decreased patient survival 122. Thus, antagonizing miRNAs likely has different consequences depending on the disease context.

Beyond determining long‐term effects, one of the greatest challenges with miRNA‐based therapeutics is finding effective delivery systems, particularly to the brain. There are various obstacles to efficient delivery including cellular resistance to the uptake of oligonucleotides, sequestration of treatments in the liver, and the blood–brain barrier, which prevents the delivery of most drugs to the brain. A recent study by Song et al. 123 addressed the first issue, finding that R3V6 peptides were able to protect anti‐miR‐21 oligonucleotides from nucleases, while delivering the treatment into the cells more efficiently than other modifications, including polyethylenimine. The peptide was also effective at reducing miR‐21 levels and inducing apoptosis in GBM cells, suggesting that the R3V6 peptide may be a useful carrier for antisense oligonucleotides 123. Other modifications have also been tested, including cholesterol, which allows for injection of antagomirs. However, cholesterol‐conjugated miRNAs are not able to access all tissues, primarily accumulating in the liver 124. Thus, much work remains to be done on optimization of delivery strategies.

#### miRNA sponges

An alternative to antisense oligonucleotides is the miRNA sponge, which contains multiple binding sites for the miRNA of interest, competitively inhibiting it from binding to its target mRNA. Importantly, the sponge's binding sites are specific to the miRNA's seed region, allowing a single sponge to block all miRNA family members containing the same seed sequence (Fig. 2A) 125.

The efficacy of miRNA sponges depends on the affinity and avidity of binding sites as well as the concentration of sponge RNA relative to miRNAs. miRNA sponges can be modified by manipulation of binding sites and their separating spacers. Binding sites can be either perfectly antisense or can contain mismatches in the middle positions, decreasing vulnerability to Ago2‐mediated endonuclease cleavage. The number of binding sites can also be altered, although increasing the number can expedite sponge RNA degradation. Further, variations in the mismatches and spacers can be introduced to reduce the risk of recombination during cloning or of introducing unintended binding motifs for other factors 125.

Chen et al. 126 demonstrated the utility of miRNA sponges in a glioma cell line and orthotopic mouse model, using an miRNA sponge to inhibit miR‐23b, which functions as an oncogene in GBM. Knockdown of miR‐23b led to a significant reduction in tumor malignancy accompanied by downregulation of HIF1*α*,* β*‐catenin, MMP2, MMP9, VEGF, and ZEB1, as well as increased expression of VHL and E‐cadherin. This was able to reduce angiogenesis, migration, and invasion of the GBM cells, thereby inhibiting cancer progression 126.

Further improvements have been made on first‐generation sponges, resulting in optimized, more potent miRNA sponges such as “tough decoy RNAs” (TuD RNA). TuD RNAs place the miRNA binding site(s) in the single‐stranded region of short stem‐loops, precisely presenting them for binding to miRNA complexes. This modification allows for more specific and efficient biological effects, including long‐term suppression of specific miRNAs 127.

There are also naturally occurring miRNA sponges, such as circular RNAs (circRNA). These RNAs are highly abundant, with thousands found in mammals 128. CircRNAs are generated via backsplicing or through partial degradation of intron lariat RNAs, forming stable closed loops that are resistant to debranching enzymes and RNA exonucleases. A circRNA sponge for miR‐7 (ciRS‐7) has been of particular interest after Hansen et al. 129 found that ciRS‐7 and miR‐7 were coexpressed in the mouse brain. ciRS‐7 contains more than 70 conserved miRNA target sites and strongly suppresses miR‐7 activity, resulting in increased levels of miR‐7 targets. As circRNAs are more thoroughly investigated, they may provide further insight for designing more effective strategies for regulating miRNAs and miRNA target genes.

#### Mimics

miRNA replacement therapy aims to restore or amplify a loss of function, particularly of tumor suppressor activity. This can be achieved through administration of synthetic miRNA mimics, which have identical sequences to their naturally occurring equivalents (Fig. 2B). For example, Chen et al. found that miR‐203 was significantly decreased in high WHO grade gliomas as compared to low WHO grade gliomas and normal brain tissue. Transfection of miR‐203 mimics into U251 human GBM cells markedly downregulated expression of phospholipase D2, a target of miR‐213 which is thought to be oncogenic in GBM. This suppressed the proliferation and invasion of U251 cells, demonstrating the utility of mimics for correcting miRNA depletion.

Mimics can also be delivered systemically with technologies used for delivering short interfering RNAs. Indeed, Ibrahim et al. 130 was able to use polyethylenimine (PEI)‐mediated delivery of chemically synthesized, unmodified miR‐145 and miR‐33a to preclinically validate the delivery method in a mouse model of colon carcinoma. Intraperitoneal injection of PEI‐complexed miR‐145 substantially reduced tumor proliferation and increased apoptosis of tumor cells, while miR‐33a decreased oncogenic kinase Pim‐1 and dampened tumor cell proliferation. Administration of both miRNAs resulted in significant reductions in tumor size, illustrating efficacy of both the mimics and the delivery mechanism.

Investigations of miRNA mimics in clinical trials have also begun. MRX34, a liposome‐formulated mimic of miR‐34 is currently undergoing a Phase I study conducted by Mirna Therapeutics. This study will examine its effects on unresectable primary liver cancer or advanced metastatic cancer with liver involvement (ClinicalTrials.gov Identifier: NCT01829971). While not targeting GBM, the expansion of miRNA use in treatment strategies represents promising advancement.

#### Additional delivery methods

As previously noted, one of the greatest challenges to efficacy of miRNA‐based therapies is the absence of effective delivery mechanisms. Currently, there are a myriad of strategies being investigated, focusing on delivery to various locations. One approach that has been gaining traction for use in gene therapy is the utilization of vectors based on adeno‐associated viruses (AAV). AAVs provide a long‐term treatment option that displays high transduction efficacy and tissue‐specific tropism 131. There are currently 12 AAV serotypes and more than 100 variants for the transfer of foreign genes into the liver, pancreas, heart, lung, skeletal muscle, and central nervous system 132. A major barrier to using AAVs in humans is the low but persistent host immune response seen in preclinical and clinical trials 133. However, there are now modified, or recombinant AAVs (rAAV), which are even more effective than the wild‐type, with lower risk and greater predictability. Currently, rAAVs are being used in clinical trials for various diseases including muscular dystrophy, Parkinson's, cystic fibrosis, and hemophilia B 133.

As gene transfer represents a long‐term strategy, the most useful miRNAs for AAV‐mediated delivery are those that are highly expressed and well tolerated in normal tissues but are lost in tumor cells. Kota et al. 134 used this approach with miR‐26a, an miRNA that is expressed at high levels in normal tissue but is lost in hepatocellular carcinoma. Systemic administration of miR‐26a via AAVs resulted in increased levels of miR‐26a in the tumors and subsequent inhibition of cancer cell proliferation, induction of tumor‐specific apoptosis, and dramatic protection from disease progression without toxicity. Thus, AAVs may represent one efficacious approach for miRNA delivery, although not appropriate for all contexts.

Nanoparticles provide another mechanism for delivery of miRNAs. Nanoparticles are highly modifiable and can be optimized for different uses by modulating the material, size, charge, and presence of surface proteins. Kouri et al. 135 used gold nanoparticles to develop a novel construct of RNA‐based spherical nucleic acids (SNA) functionalized with mature miR‐182 sequences (182‐SNA). SNAs exhibit high cellular uptake, high stability, high resistance to nuclease degradation, low activation of the innate immune system, and no significant acute or long‐term toxicity in animal models. SNAs also display preferential accumulation within intracerebral gliomas due to the sequestration of nanomaterials in tumors because of the abnormal architecture of tumor blood vessels. 182‐SNAs were able to effectively cross the blood–brain and blood–tumor barrier following systemic intravenous administration in glioma‐bearing mice. The constructs disseminated throughout the glioma parenchyma where glioma cells displayed robust cellular uptake of the 182‐SNAs. This was accompanied by potent downregulation of target proteins as well as enhanced sensitivity toward chemotherapy‐induced apoptosis, resulting in significantly reduced tumor burden and increased survival time with no significant adverse side effects 135, 136.

Mesenchymal stem cells are also predicted to be effective delivery vehicles due to their ability to preferentially home to tumor tissue. Lee et al. 137 found that MSCs derived from bone marrow, adipose tissue, placenta, and umbilical cords could deliver synthetic miRNA mimics to glioma cells through both gap junction‐dependent and ‐independent mechanisms. MSCs transfected with Cy‐3‐labeled miR‐124 and miR‐145 efficiently delivered the miRNAs to adjacent cocultured glioma cells. This resulted in downregulation of miR‐124 and miR‐145 target genes and decreased the migration and self‐renewal capacities of glioma cells 137. Munoz et al. 138 likewise confirmed that MSCs could transfer miRNA‐based therapies to GBM cells. They found that anti‐miR‐9 was transferred from MSCs to GBM cells via gap junctional intercellular communication as well as through the release of microvesicles. The delivery of anti‐miR‐9 sensitized GBM cells to TMZ, increasing caspase activity and cell death 138. Further, studies have demonstrated that MSCs are capable of localizing to endogenous high‐grade gliomas after intraarterial delivery in mice 139.

### Challenges

Despite advances in understanding of both miRNAs and GBM, there are still major difficulties to overcome before widespread application of miRNA‐based treatments becomes feasible. The lack of an effective delivery system with enough specificity and efficacy is one of the greatest challenges in utilizing miRNAs for therapeutics. This is particularly relevant for GBM, where both the blood–brain and blood–tumor barriers prevent the accumulation of therapeutic drug concentrations in brain tumors. However, with the development of more accurate in vitro models of the blood–brain barrier, we have the opportunity to more rapidly and easily screen new delivery mechanisms for potential in penetrating the blood–brain barrier and specifically targeting tumor sites 140. Alternatively, emphasis on the development of more effective local delivery techniques that can bypass the blood–brain barrier could also offer more efficacious solutions 141.

Additional challenges could arise from the ability of miRNAs to engage with multiple targets. While offering many advantages, this ability is a double‐edged sword. The potential for widespread effects increases the risk of deleterious repercussions from impact on unintended targets. Additionally, as mRNAs can be targeted by numerous different miRNAs, compensation mechanisms through alternative pathways could compromise treatment outcomes.

Finally, tumor heterogeneity is a major challenge as there are substantial variations across individuals as well as between different cells within tumors. This includes the presence of GSCs, which can dramatically impact tumor aggressiveness and resistance to treatments. Thus, each tumor may respond differently to the same therapeutic strategies, necessitating a need for more individualized treatment options. With the increasing ease in obtaining genomic information for each patient, miRNA profiling could be used as a critical factor for evaluating response to treatment. Thus, more comprehensive characterization of potential predictive and prognostic miRNA markers is essential to optimizing personalized treatments for GBM.

## Conclusion

miRNAs have been called “small RNA molecules with a huge impact” due to their wide range of targets and functions in carcinogenesis 142. The understanding of miRNAs is rapidly developing with potential to drastically change the conceptualization of developmental biology, including the development of pathological conditions such as cancer. In particular, miRNA expression profiling has enhanced our understanding of GBM disease progression, providing valuable information on pathogenesis and potential targets. Detecting and quantifying miRNAs in tissue and serum will likely become a standard diagnostic and prognostic tool for GBM, potentially serving as a mechanism for creating personalized treatment strategies. As the role of miRNAs in GBM become more well‐understood and novel delivery methods are developed and optimized, miRNA‐based targeted therapies could provide a promising advancement in cancer therapeutics. Thus, mapping of expression profiles for different disease states as well as identification of pathways targeted by miRNAs could be a critical step forward in determining more effective treatment mechanisms for various cancers, including GBM.

## Conflict of Interest

The authors declare that they have no conflict of interest.
